# Genome-Wide Identification and Functional Characterization of the Cation Proton Antiporter (CPA) Family Related to Salt Stress Response in Radish (*Raphanus sativus* L.)

**DOI:** 10.3390/ijms21218262

**Published:** 2020-11-04

**Authors:** Yan Wang, Jiali Ying, Yang Zhang, Liang Xu, Wanting Zhang, Meng Ni, Yuelin Zhu, Liwang Liu

**Affiliations:** National Key Laboratory of Crop Genetics and Germplasm Enhancement, Key Laboratory of Horticultural Crop Biology and Genetic Improvement (East China) of MOAR, College of Horticulture, Nanjing Agricultural University, Nanjing 210095, China; wangyanhs@njau.edu.cn (Y.W.); 2019204031@njau.edu.cn (J.Y.); radishlab@njau.edu.cn (Y.Z.); nauxuliang@njau.edu.cn (L.X.); 2018104061@njau.edu.cn (W.Z.); 2019104066@njau.edu.cn (M.N.)

**Keywords:** *CPA* gene family, *RsNHX1*, over-expression, virus-induced gene silence, salt resistance, radish

## Abstract

The CPA (cation proton antiporter) family plays an essential role during plant stress tolerance by regulating ionic and pH homeostasis of the cell. Radish fleshy roots are susceptible to abiotic stress during growth and development, especially salt stress. To date, *CPA* family genes have not yet been identified in radish and the biological functions remain unclear. In this study, 60 *CPA* candidate genes in radish were identified on the whole genome level, which were divided into three subfamilies including the Na^+^/H^+^ exchanger (NHX), K^+^ efflux antiporter (KEA), and cation/H^+^ exchanger (CHX) families. In total, 58 of the 60 *RsCPA* genes were localized to the nine chromosomes. RNA-seq. data showed that 60 *RsCPA* genes had various expression levels in the leaves, roots, cortex, cambium, and xylem at different development stages, as well as under different abiotic stresses. RT–qPCR analysis indicated that all nine *RsNHXs* genes showed up regulated trends after 250 mM NaCl exposure at 3, 6, 12, and 24h. The *RsCPA31 (RsNHX1)* gene, which might be the most important members of the RsNHX subfamily, exhibited obvious increased expression levels during 24h salt stress treatment. Heterologous over-and inhibited-expression of *RsNHX1* in *Arabidopsis* showed that *RsNHX1* had a positive function in salt tolerance. Furthermore, a turnip yellow mosaic virus (TYMV)-induced gene silence (VIGS) system was firstly used to functionally characterize the candidate gene in radish, which showed that plant with the silence of endogenous *RsNHX1* was more susceptible to the salt stress. According to our results we provide insights into the complexity of the *RsCPA* gene family and a valuable resource to explore the potential functions of *RsCPA* genes in radish.

## 1. Introduction

Plants respond to salt stress by regulating the cell ion and pH balance through a variety of mechanisms, which are mainly dependent on ion transporters in cell membranes and organelle membranes [1,2]. Cation proton antiporters (CPAs) are mainly involved in the exchange and transport of monovalent cations in plants, which not only reverses the transport of Na^+^ with H^+^ but also exchanges and transports monovalent cations, such as K^+^ and Li^+^ [3,4,5]. The CPA family is divided into two main superfamilies, named CPA1 and CPA2 [6]. The CPA1 superfamily contains the Na^+^/H^+^ exchanger family (NHX), which is predicted to have 10–12 membrane-spanning domains in plants and it has been confirmed with an important effect on salt tolerance of plants [2,7]. CPA2 consists of the K^+^ efflux reverse transporter family (KEA) and cation/H^+^ exchanger family (CHX), which is predicted to have 8–14 transmembrane domains [2,7], all of which contain the Na^+^/H^+^ exchanger domain (PF00999) [8]. In addition, CPA proteins are mainly localized on the plasma, vacuole membrane and organelle membrane of plant cells [2,9], which can play essential roles in plants responding to environmental stress and maintaining the homeostasis of pH and ions [8,10].

Identification and characterization of the CPA family has been extensively reported in several plant species, including *Arabidopsis thaliana* [11], rice (*Oryza sativa*) [12], grape (*Vitis vinifera*) [13], and pear (*Pyrus bretschneideri*) [14]. In recent years, increasing evidence has indicated that some *CPA*s, especially *NHX*s, respond to salt stress, cell expansion, pH and ion balance regulation, osmotic regulation, and vesicle transport, as well as protein processing and floral organ development [15,16,17,18,19]. The previous studies showed that the NHX subfamily consisted of eight members (AtNHX1–AtNHX8) in *Arabidopsis*, which were mainly involved in the exchange and transport of Na^+^ and H^+^ [20,21,22]. Recent reports further revealed that the *NHX1* gene in *Vigna unguiculate*, *Malus domestica*, *Sesuvium portulacastrum*, *Arachis hypogaea*, and *Helianthus tuberosus* can regulate salt tolerance [12,23,24,25,26]. For example, *VuNHX1* displayed higher salt tolerance through over-expression in *Arabidopsis* [23]. Over-expression of *SpNHX1* yeast cells grew better and accumulated more Na^+^ than control, indicating that *SpNHX1* was a key response gene to salt stress [25]. Furthermore, over-expression of *AtNHX7* (*AtSOS1*) limited Na^+^ accumulation in xylem and stem, and improved salt tolerance in transgenic *Arabidopsis thaliana* [27]. Interestingly, the *AtCHX17* mutant accumulated less K^+^ than the control in salt stress and K^+^ deficient environment, indicating that the *AtCHX* gene was involved in the exchange and transport of K^+^ rather than Na^+^ [28]. In addition, K^+^/H^+^ reverse transporters AtKEA1–AtKEA3 played an important role in chloroplast function, osmoregulation, photosynthesis, and pH regulation [29,30].

Radish (*Raphanus sativus* L.) belongs to the Brassicaceae family and is one of the most economically important annual or biennial root vegetable crops, which is widely cultivated all over the world with high nutritional and medicinal value. The fleshy taproot is the edible organ of the radish, which has different sensitivities to salt stress [31,32]. Although some *CPA* genes have been proved to be related to salt stress response in other plant species, such as *Arabidopsis*, pear (*Pyrus bretschneideri*), grape (*Vitis vinifera*) and *Helianthus tuberosus*, the information of *CPA* genes identification at the whole genome level in radish is still limited [11,12,13,14]. The radish genomes were released to provide a helpful resource to identify the *CPA* gene family at the whole genome level [33]. Our study aimed to systematically identify CPA family members from the radish genome, map *RsCPAs* onto chromosomes, and investigate gene structure and conserved motifs. Moreover, the expression patterns of *RsCPAs* in different developmental stages and tissues were analyzed and also explored for differentially sensitive genes under abiotic stress, especially in salt stress. Furthermore, the biological function of *RsNHX1* was validated for heterologous over-expression and inhibited-expression, as well as through turnip yellow mosaic virus (TYMV)-mediated silencing (VIGS) in radish. The outcomes of our study lay the foundation for further characterization of these *CPA* genes for roles in radish salt-tolerance processes.

## 2. Results

### 2.1. Identification and Classification of RsCPA Members in Radish

The Hidden Markov Model (HMM) profile was firstly performed to search the whole genome protein sequence of radish with the Na^+^/H^+^ exchanger domain (PF00999), and a total of 61 putative CPA proteins were obtained. Following, the CDD and InterPro tools were employed for detecting the completeness of the Na^+^/H^+^ exchanger domain and then one was excluded. Finally, 60 non-redundant and complete RsCPA members were identified among the radish genome, which were correspondingly named as RsCPA01–RsCPA60 (Table S1).

Through the physical and chemical properties analysis, the protein sizes of RsCPA ranged from 231 to 1172 amino acids (AAs) with molecular weight (MWs) from 25.97 to 126.11 kDa and the theoretical isoelectric point (pI) varied from 4.98 to 9.21. In addition, the instability coefficient reached from 27.56 to 46.90 and 42 members were <40.00, which were considered as stable proteins. The aliphatic index varied from 95.31 to 127.56, indicating that most RsCPA proteins contained a lot of aliphatic amino acids. The grand average of hydropathicity (GRAVY) ranged from 0.048 to 0.798, suggesting that all RsCPA proteins were hydrophobic proteins (Table S1).

### 2.2. Phylogenetic Analysis of RsCPA Members

**Figure 1:**
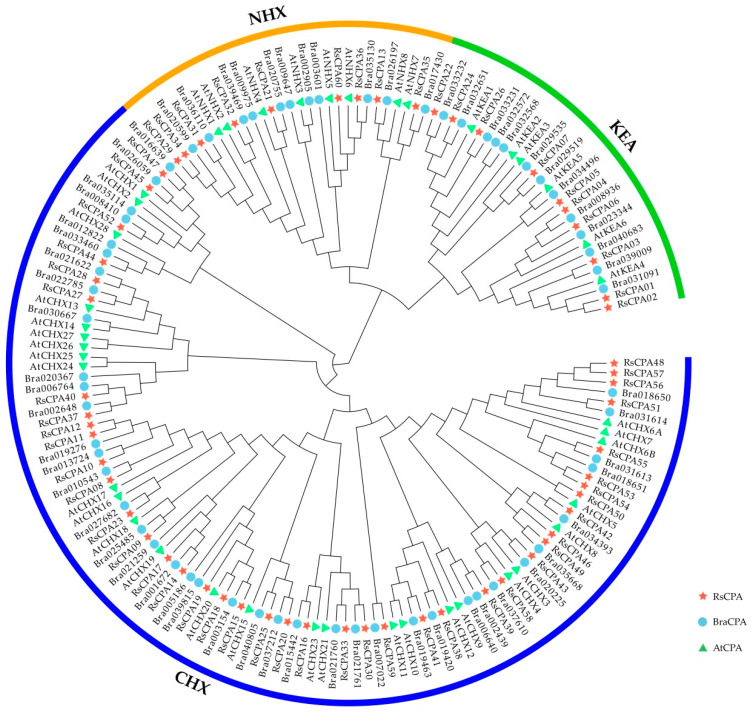
Phylogenetic relationship of RsCPA, BraCPA, and AtCPA members.

To investigate the classification of the CPA subfamily of radish and the evolutionary relationship with other species, full-length CPA protein sequences of radish, *Arabidopsis* and *Brassica rapa* were extracted and aligned to construct a neighbor-joining (NJ) phylogenetic tree (Figure 1). A total of 166 CPA protein members in these three species (containing 60 radish, 42 *Arabidopsis*, and 64 *Brassica rapa*) were categorized into three subfamilies, namely NHX, KEA, and CHX. Among them, the NHX group had 28 members containing nine, eight, and 11 members of radish (15%), *Arabidopsis* (19.05%), and *Brassica rapa* (17.19%), respectively. The KEA group included 29 members with ten, six, and 13 members of radish (16.67%), *Arabidopsis* (14.29%), and *Brassica rapa* (20.31%), respectively. The CHX group was the most abundant subfamily and had 109 members with 41, 28, and 40 members in radish (68.33%), *Arabidopsis* (66.67%), and *Brassica rapa* (62.5%), respectively (Table 1). The phylogenetic relationships indicated that the CPA proteins in radish had stronger homology with *Brassica rapa* than *Arabidopsis*.

Meanwhile, compared with several dicotyledon and monocotyledon crops, the number of CPA gene in radish was closer to *Malus domestica* and *Pyrus bretschneideri*, while it was significantly different from that in *Prunus persica*, *Fragaria vesca*, *Prunus mume*, and *Vitis vinifera*. Especially compared with monocotyledonous plants, such as *Oryza sativa*, *Zea mays*, and *Sorghum bicolor*, the difference of CPA gene numbers was very significant (Table 1).

### 2.3. Gene Structure and Motif Composition Analysis

**Figure 2:**
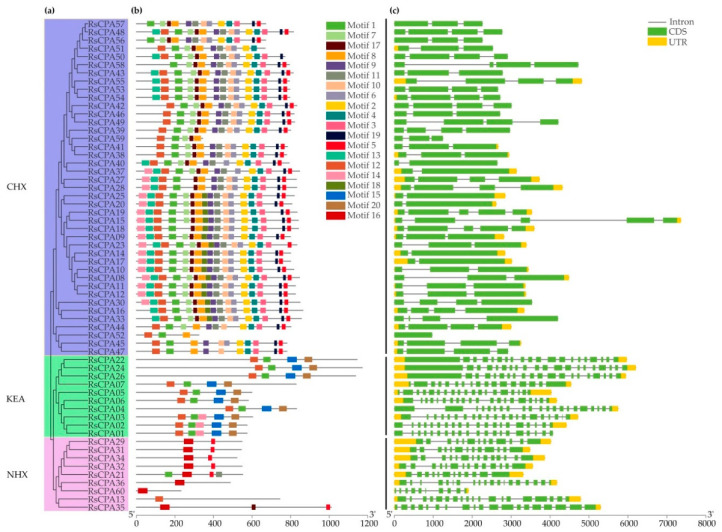
Conserved motif and gene structure distribution of RsCPA proteins. (**a**) Phylogenetic tree of RsCPA proteins. The scale bar indicates 200 aa; (**b**) Conserved motif distribution of RsCPA proteins; (**c**) Exon-intron structure of *CPA* genes in radish. The scale bar indicates 1 kb.

All the 60 RsCPA members were divided into three subfamilies, including 9 RsNHXs, 10 RsKEAs, and 41 RsCHXs (Figure 2a). The distributions of RsCPA protein motifs were conducted by Multiple Em for Motif Elicitation (MEME) and 20 conserved motifs were generated (Figure 2b, Figure S1). Most RsCPA members in the same subfamily had similar motif compositions, suggesting that these proteins might have conservative functions. Among them, motif 1 and 12 were found in the CHX, KEA, and NHX subfamilies, indicating that were highly conserved in all RsCPA proteins. Additionally, some motifs were distributed in two subfamilies. For instance, motif 5, 11, and 17 were distributed in the CHX and NHX subfamilies, while motif 14 was distributed in the CHX and KEA subfamilies. Intriguingly, several motifs were only detected in specific RsCPA subfamilies. For example, the RsCHX subfamily independently contained diverse motifs, such as motif 3, 4, 6, 7, 8, 10, 13, 18, and 19. However, the KEA subfamily exclusively contained the motif 15 and 20, as well as the RsNHX subfamily that specifically contained motif 16 (Figure 2b).

Furthermore, exon-intron analysis was investigated to obtain the structure information of *RsCPA* genes. As shown in Figure 2c, the gene structures involved in the same subfamily were similar, while the lengths of the exon and intron were different. Compared with the *RsCHXs* subfamily, the gene structures of *RsNHXs* and *RsKEAs* were more complex. Among them, the exon numbers of *RsCHXs* were generally one to five. However, the exon numbers of *RsNHXs* and *RsKEAs* ranged from 10 to 19 and 17 to 21, respectively. Additionally, all members in *RsNHX* subfamily as well as most *RsKEA* ones (except *RsCPA01*) had UTR (untranslated region), whereas several members of the *RsCHX* subfamily had no UTR region (Figure 2c).

### 2.4. Promoter Elements and Transmembrane Region Analysis

**Figure 3:**
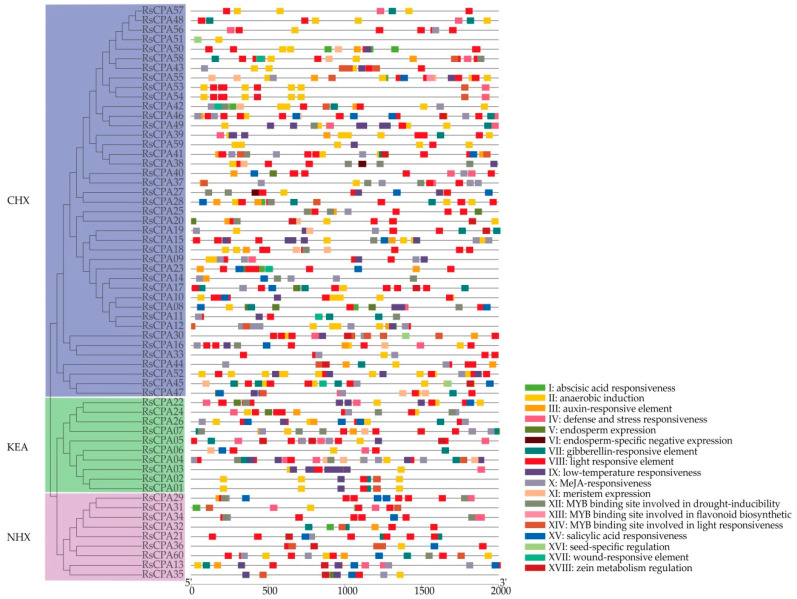
*Cis*-acting elements on promoters of *RsCPA* genes. Distribution of *cis*-acting elements on promoters of *RsCPA* genes.

The putative promoter sequence (2000 bp upstream region of transcription initiation site) of *RsCPA* genes was submitted to PlantCARE to search for *cis*-acting elements. A total of 103 *cis*-acting elements were identified. Except basic promoter elements, such as the CAAT box and TATA box, 18 other important *cis*-acting elements related to plant growth and development and various stresses were explored. In Figure 3, it was shown that the distribution pattern of *cis*-acting elements were diverse, indicating that the expression of *RsCPA* genes might be regulated by various factors. In the aspect of hormone regulation, Abscisic Acid (ABA), auxin, Gibberellin A_3_ (GA_3_), and Methyl Jasmonate (MeJA) responsiveness elements frequently existed. While in terms of stress, anaerobic induction, defense and stress, low temperature, and wound-responsive elements were resided. Moreover, the zein metabolism regulation element existed in 14 promoters of *RsCPA* genes, suggesting that *RsCPA* genes might participate in the process of metabolic regulation. Intriguingly, there were 12 promoters of the *RsCPA* gene involved in endosperm expression, among them, *RsCPA27* and *RsCPA38* participated in endosperm specific negative expression (Figure 3 and Figure S2). Additionally, the transmembrane regions of RsCPA proteins showed that all RsCPA proteins, except RsCPA07, contained transmembrane regions that varied from three to 14 (Table S2).

Futhermore, an interaction of CPA orthologs co-regulatory network was constructed based on the stress-inducible RsCPA orthologs in *Arabidopsis* (Figure S3). It was found that the combination score of the ATCHX1 and KEA4 protein was highest at 0.868, suggesting that it was involved in a stronger relationship between some specific RsCPA proteins, such as RsCPA45, RsCPA47, RsCPA03, RsCPA01, and RsCPA02.

### 2.5. Chromosomal Localization and Gene Distribution Analysis

**Figure 4:**
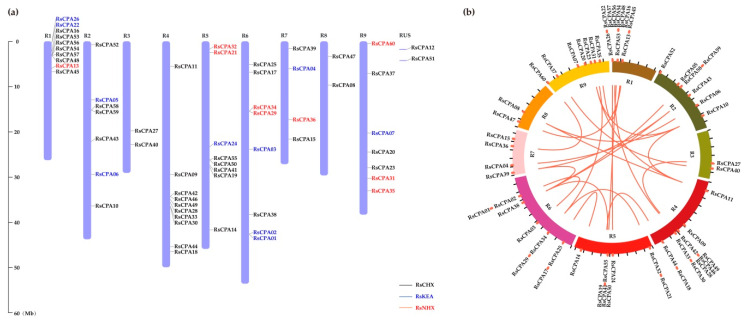
Chromosomal distribution and chromosomal relationships of *RsCPA* genes. (**a**) Chromosomal distribution of *RsCPA* genes; (**b**) Genome distribution and collinearity of the RsCPA family. Red lines indicate the collinear relationship among genes.

A total of 58 *RsCPA* genes (96.67%) were successfully mapped to the R1–R9 chromosomes of radish by TBtools, except *RsCPA12* and *RsCPA51* (Figure 4a). R1 and R4 harbored the most *RsCPA* genes (Ten, 17.67%), followed by R5 and R6 (Eight, 13.37%), while R3 and R8 contained the least *RsCPA* genes (Two, 3.33%). Genome duplication events have facilitated the expansion of plant gene families, including whole-genome duplication (WGD)/segmental duplication, dispersed duplication (DD), tandem duplication (TD), proximal duplication (PD), and transposed duplication (TRD) [34,35,36]. The duplication types driving expansion of the *RsCPA* gene family was explored by Multiple Collinearity Scan toolkit (MCScanX). Each *RsCPA* gene was mapped on the radish genome based on the position coordinates to deduce the evolutionary relationship (Figure 4b). Totally, 24 pairs of *CPA* genes in radish had collinear relationships. Of these, 36 (60%) *RsCPA* genes were duplicated and retained in the WGD event, indicating that the WGD/segmental duplication type played an important role in expansion of the *RsCPA* gene family.

### 2.6. Evolution Analysis of the RsCPA Genes

**Figure 5:**
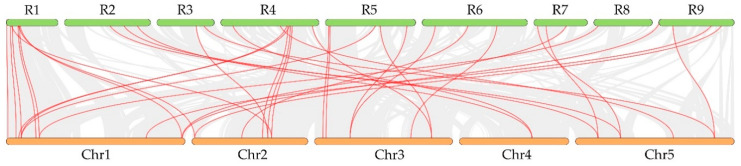
Evolution analysis of the *RsCPA* gene family. Synteny blocks of *CPA* genes between radish and *Arabidopsis*. Colored lines connecting two chromosomal regions indicate syntenic regions between *Arabidopsis* (Chr1–5) and radish (R1–9) chromosomes.

The possible evolution mechanism of the RsCPA family was investigated by using synteny blocks. To further infer the origin and evolutionary history of CPA members, the synthetic regions between radish and *Arabidopsis* were analyzed and compared (Figure 5). According to the synthetic map, there were 57 pairs of collinear genes in radish and *Arabidopsis*, containing 12 *NHXs*, six *KEAs*, and 39 *CHXs*. Five pairs of syntenic orthologous genes (one to one) were identified, including *RsCPA06AtKEA6*, *RsCPA20*–*AtCHX15*, *RsCPA26*–*AtKEA2*, *RsCPA44*–*AtCHX28*, and *RsCPA45*–*AtCHX2*. These genes could be traced back to a common ancestor in *Arabidopsis* and radish. Among the two synthetic orthologous gene pairs, one radish gene corresponded to multiple *Arabidopsis* genes, such as *RsCPA26*–*AtKEA1/2*, *RsCPA27*–*AtCHX13/14/21/25/26*, and *RsCPA32*–*AtNHX1/2/6*. Accordingly, there also existed syntenic orthologous gene pairs with one *Arabidopsis* gene corresponding to multiple radish genes, for instance, *AtCHX6B*–*RsCPA39/42/43/53/55*, *AtNHX6*–*RsCPA32/36/60*, *AtKEA1*–*RsCPA24/26*, among others. In addition, the gene pairs of two or three *Arabidopsis* genes corresponding to the same two radish gene pairs were also found, containing *AtCHX3/4/6B*–*RsCPA39/43*, *AtCHX13/14/26*–*RsCPA27/28*, *AtCHX6B/7*–*RsCPA42/53*, and *AtNHX1/2*–*RsCPA31/32*. A series of synteny events indicated that many *CPA* genes appeared before the divergence of the *Arabidopsis* and radish lineages (Figure 5, Table S3).

The non-synonymous/synonymous substitution ratio (Ka/Ks) for the 24 gene pairs was calculated to determine the selection pressure among duplicated *RsCPA* genes. Most of the *RsCPA* duplication genes (except *RsCPA11*–*RsCPA23* and *RsCPA21*–*RsCPA34*) had a Ka/Ks < 1, indicating that they had experienced strong purifying selective pressure (Table S4).

### 2.7. Spatial and Temporal Expression Profiles of RsCPA Genes

**Figure 6:**
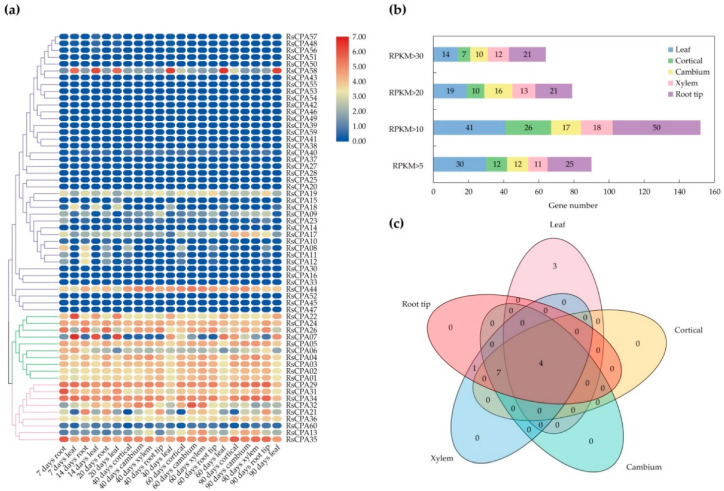
Expression profile of *RsCPA* genes in different stages and tissues. (**a**) *RsCPA* genes expression heatmap in six stages (7, 14, 20, 40, 60, and 90 days) and five tissues (cortical, cambium, xylem, root tip, and leaf). Expression values were calculated by (reads per kilobase per million) RPKM. The scale represents relative expression values; (**b**) Number of *RsCPAs* with a high transcriptional abundance level (RPKM > 10) in each tissue; (**c**) Venn diagram of overlapping *RsCPAs* that are abundantly expressed (RPKM > 10) in different tissues.

According to the reads per kilobase per million (RPKM) values, the heatmap was generated to characterize the divergence in expression patterns of *RsCPA* genes among special tissues (cortical, cambium, xylem, root tip, and leaf) and different development stages (40, 60, and 90 d) (Figure 6). In general, the RPKM value varied from 0 to 103.06 and all *RsCPA* genes exhibited diverse expression patterns. Most of the *RsNHX* and *RsKEA* genes showed high expression levels among the five tissues, while the *RsCHX* genes had extremely low expression or were hardly expressed, as well as a few that showed tissue-specific expression (Figure 6a). It was found that *RsCPA58* (*RsCHX*) was only highly expressed in leaves rather than other tissues, indicating that it might be a leaf-specific gene. Moreover, *RsCPA35* was highly expressed in roots after 7 days, while it was down-regulated in roots at other development stages, suggesting it might be a spatiotemporal-specific gene. Furthermore, 68.33, 43.33, 28.33, 30, and 83.33% of the *RsCPA* genes showed a higher transcriptional abundance value (RPKM value > 10) in the five tissues, respectively (Figure 6b). Notably, *RsCPA24*, *RsCPA29*, *RsCPA34*, and *RsCPA35* had abundant expression (RPKM value > 10) in five tissues (Figure 6c). These *RsCPA* genes might play various functions in the development of different tissues during various stages.

### 2.8. The RsCPA Genes Expression Levels under Abotic Stresses

Based on our previous RNA-Seq. data in radish taproots, the differential expression levels of *RsCPA* genes under various abiotic stresses were investigated, including heavy metal (HM, such as Cadmium (Cd), Chromium (Cr), and lead (Pb), temperature, and salt exposure. As a result, a total of 35, 38, 35, 33, and 29 *RsCPA* genes were differentially expressed during Cd, Pb, Cr, heat, and salt stress (Fold change >1, *p*-value < 0.05), respectively (Figure S4). For instance, *RsCPA13* was up-regulated in response to Cd, Pb, Cr, and salt stresses, while it was down-regulated under high temperatures. *RsCPA08* was up-regulated in response to Cd and Pb, whereas it was down-regulated under Cr and salt stress. Additionally, *RsCPA35* was up-regulated in response to Cd, Pb, high temperature, and salt stresses, but it was down-regulated under Cr stress.

Furthermore, RT–qPCR was conducted to explore the expression levels of *RsNHXs* subfamily genes under the stress of salt exposure. On the whole, all of the *RsNHX* genes were significantly up-regulated under the 250 mM salt stress treatment. The expression level of *RsCPA13*, *RsCPA29*, *RsCPA31*, and *RsCPA35* were highly increased during the 24 h salt exposure. Moreover, several genes were significantly up-regulated after 12 h salt exposure, such as *RsCPA21* and *RsCPA34*. Notably, the expression level of *RsNHXs* recovered to normal levels after 96 h salt exposure, implying that *RsNHXs* might play a crucial role in the process of salt stress response (Figure S5).

### 2.9. Ectopic Expression of the RsNHX1 Gene in Arabidopsis Can Influence Salt Tolerance

**Figure 7:**
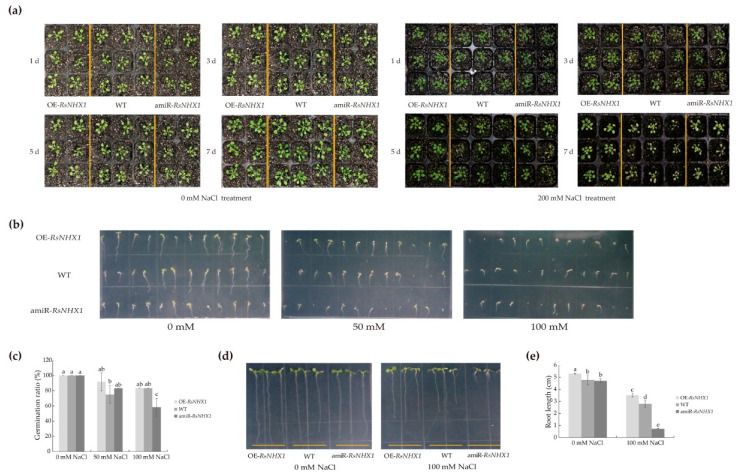
Phenotypic identification of over-expression and inhibit-expression *RsNHX1* transgenic lines responding to salt stress in *Arabidopsis*. (**a**) Morphological comparison between wild-type (WT), OE–*RsNHX1* and amiR–*RsNHX1* transgenic lines; (**b**) Morphological comparison of germination between WT, OE–*RsNHX1* and amiR–*RsNHX1* transgenic seedlings with different concentrations of NaCl; (**c**) Statistical analysis of germination ratio after salt stress. Each bar shows the mean ± SD of the double assay. Values with different letters indicate significant differences at *p* < 0.05 according to Duncan’s multiple range tests; (**d**) Morphological comparison of root length between WT, OE–*RsNHX1*, and amiR–*RsNHX1* transgenic seedlings with different concentrations of NaCl; (e) Statistical analysis of root length after salt stress. Each bar shows the mean ± SD of the triplicate assay. Values with different letters indicate a significant difference at *p* < 0.05 according to Duncan’s multiple range tests.

To confirm the biologic function of *RsNHX1* gene in the salt stress response of plant, over-expression (OE–*RsNHX1*) and amiRNA-induced inhibit-expression (amiR–*RsNHX1*) constructs were introduced into wild-type *Arabidopsis* (WT). Four and six independent transgenic lines were respectively generated for OE–*RsNHX1* (#2, 4, 5 and 6) and amiR–*RsNHX1* vectors (#1, 2, 3, 4, 5, and 6) (Figure S6). Following, each of the six OE–*RsNHX1* and amiR–*RsNHX1* transgenic lines were compared with nine WT lines under the 200 mM NaCl stress. It was found that the OE–*RsNHX1* transgenic lines were slightly yellowed and grew better than the control lines, while most of the amiR–*RsNHX1* transgenic lines turned markedly yellow and their growth was inferior to control lines (Figure 7a). For salt stress assays at the seedling stage, the germination ratio of two transgenic seedlings and WT seedlings were counted under 0, and 100 mM NaCl treatment (Figure 7b). As shown in Figure 7b,c, the germination rate of OE–*RsNHX1* seedlings treated with 50mM NaCl was higher than the WT, while amiR–*RsNHX1* seedlings treated with 100 mM NaCl was significantly inferior to the WT. Furthermore, the root length of transgenic seedlings and the WT were measured under 0 and 100 mM NaCl treatment (Figure 7d). The root of OE–*RsNHX1* seedlings exhibited continuing elongation, whereas the amiR–*RsNHX1* seedlings were significantly inhibited under 100 mM NaCl exposure (Figure 7e). These results indicated that *RsNHX1* might play a positive role in the salt tolerance of radish. 

### 2.10. Functional Analysis of RsNHX1 in Radish Confirms that It Can Positively Regulates Salt Tolerance

**Figure 8:**
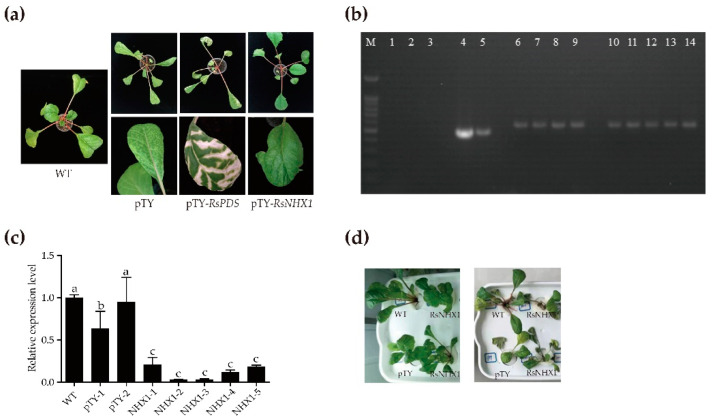
Functional analysis of *RsNHX1* gene in radish during the salt stress. (**a**) Phenotype of virus symptoms on leaves of the entire plant; (**b**) Electrophoresis identification of pTY–S. M–marker, WT (*line 1–3*), pTY–S (*line 4* and *5*), pTY–*RsPDS* (*line 6–9*), pTY–*RsNHX1* (*line 10–14*); (**c**) Relative expression levels of the *RsNHX*1 gene. The data represented are means of the triplicate assay and error bars represent the standard deviation of means. Different letters above bars indicate significant differences (*p* < 0.05) between plants; (**d**) Phenotype of plants after salt stress for seven days.

We further identify the function of *RsNHX1* gene responding to salt stress in radish by using turnip yellow mosaic virus (TYMV)-induced gene silencing (VIGS) technique to silence *RsNHX1* expression. Phytoene desaturase (PDS) was employed as a reporter gene to test whether the TYMV–derived vector can silence the endogenous gene of radish. Plants treated with the pTY–S virus vector designed to silence *RsNHX1* expression (pTY–*RsNHX1*), with the wide type (WT), empty pTY–S silencing vector as well as the pTY–*RsPDS* gene served as mock, empty vector, and positive controls, respectively. Three weeks after particle gun bombardment in radish seedlings, typical phenotype of chlorophyll photobleaching and TYMV spots were separately observed on the leaves of pTY–*RsPDS*, pTY–*RsNHX1*, and pTY–S plants, indicating that TYMV–VIGS system was effective in radish (Figure 8a).

The total RNA was extracted from the diseased leaves of the suspected radish. Gel electrophoresis showed that the 488 bp PCR products were amplified in two pTY–S plants, the 522bp PCR products were amplified in four pTY*–RsPDS* plants and five pTY*–RsNHX1* plants, indicating that they were successfully silenced in radish plants (Figure 8b). Furthermore, RT–qPCR revealed that the expression of positive pTY*–RsNHX1* was significantly decreased compared to the WT and positive pTY–S plants (Figure 8c). Subsequently, the positive pTY*–RsNHX1* plants showed more severe yellowing and wilting than positive pTY–S plants after 7 days of 250mM NaCl exposure (Figure 8d), showing that *RsNHX1* might be a salt sensitive gene.

## 3. Discussion

### 3.1. Genome-Wide Identification and Phylogenetic Analysis of CPA Genes in Radish

The *CPA* gene encoded a conserved Na^+^/H^+^ domain and played an important role in diverse biology processes, including salt stress, cell expansion, pH and ion balance regulation, osmotic regulation, vesicle transport, protein processing, and floral organ development [15,16,17,18,19]. With the completion of genome sequencing in many plant species, CPA family members have been reported in various plant species, including model plant *Arabidopsis* (42) and *Oryza sativa* (30), as well as several horticulture plants, such as *Vitis vinifera* (29) and *Pyrus bretschneideri* (53) [11,12,13,14]. Herein, 60 *RsCPA* members were identified from the radish genome, which showed more members than other reported species indicating that the *CPA* gene family in radish may be duplicated and expanded.

Phylogenetic analysis indicated that a total of 166 CPAs among the three species containing radish, *Brassica rapa*, and *Arabidopsis* were divided into three subfamilies: CHX (109, 65.66%), KEA (29, 17.47%), and NHX (28, 16.87%), which was largely consistent with previous studies in *Arabidopsis*, *Vitis vinifera*, and *Pyrus bretschneideri* [11,12,13,14]. Through the phylogenetic relationships analysis, it was showed that RsCPAs exhibited closer relations to BraCPAs than AtCPAs, demonstrating that the CPA proteins in radish had stronger homology with the *Brassica rapa* rather than *Arabidopsis* (Figure 1). Furthermore, gene structure and motif analysis indicated that the RsCPAs family harbored similar exon–intron structure and shared motif composition with other species, such as *Vitis vinifera* and *Pyrus bretschneideri* [13,14] (Figure 2). 

### 3.2. Evolutionary Characterization of the RsCPA Family

The expansion of the gene family was mainly caused by gene duplication [34,35]. In the process of plant evolution, duplicated genes could obtain new functions or segment existing functions to improve the adaptability of plants [34]. For instance, expansion of the *RsHSF* gene family was primarily driven by WGD or segmental duplication, which might be largely related with gene duplication [36]. It was previously reported that WGD and PDs event were mainly involved in the expansion of the CPA family in *Pyrus bretschneideri* [14]. In the present study, the predicted gene duplication was also found in radish among the *CPA* genes, indicating that the WGD event played an important role in the expansion of the *CPA* gene family (Figure 4b). In addition, Ka/Ks could be used to identify whether selective pressure existed on the *RsCPA* gene family, including positive, negative, and neutral selection. Herein, except *RsCPA11*–*RsCPA23* and *RsCPA21*–*RsCPA34*, all of the *RsCPA* duplication genes displayed a Ka/Ks < 1, suggesting that they had experienced strong purifying selective pressure. A similar Ka/Ks was also reported in the *Pyrus bretschneideri* and cotton, further confirming that the evolutionary pattern of *CPA* genes was very conservative (Table S4) [14,37].

Combined with evolutionary classification and synteny analysis, a large number of the *RsCPAs* were identified as orthologous genes in *Arabidopsis*. For example, eleven pairs seemed to be single radish-to-*Arabidopsis* pairs, presuming that these genes might exist in the genome of the last common ancestor of the two species. There also existed more complex relationships, such as single radish and multiple *Arabidopsis* genes, one *Arabidopsis* and multiple radish genes, and two *Arabidopsis* and multiple radish genes, etc. This phenomenon was similar to the evolutionary relationship of *bZIP* genes in radish and *Arabidopsis* [38]. The close relationship of orthologous genes may exhibit similar functions in different species. For instance, *AtNHX1* gene was reported high salt sensitivity [24], accordingly in our study its orthologues comparising *RsCPA31*, *RsCPA32*, and *RsCPA34* also exhibited high induction under 250 Mm NaCl salt treatments (Figure S5). The collinearity-orthologues analysis of radish and *Arabidopsis* could provide a valuable reference for further exploring the functions of these highly homologous genes in radish.

### 3.3. Roles of RsCPA Genes in Response to Different Abiotic Stresses 

Increasing evidences indicated that *CPA* genes played vital roles in a variety of abiotic stresses, including HM, temperature, and salt exposure [39,40,41,42]. For instance, *AtCHX17* mutant accumulated less K^+^ than wide type under the salt stress and K^+^ deficiency environment, indicating that AtCHX17 was involved in the absorption and transport of K^+^ [28]. Among the *CPA* genes in other plants, it was *NHX1* that could decrease the salt-tolerance ability of kallar grass at the concentration of 100 and 150 μM cadmium concentrations [43]. Moreover, *NHX1* regulated Cd^2+^ and H^+^ flow during short-term Cd^2+^ shock and confirmed that it could enhance tolerance during Cd^2+^ stress [44]. Recent studies have shown that *NHX2* homologues had a high expression under salinity stress at higher time intervals in *G. barbadense* and *G. hirsutum* [45]. The expressions of *NHX* were up-regulated in root tissues of wheat under salinity stress [46]. In this study, the transcriptome data of the radish taproot showed that nearly one-half of *RsCPA* genes displayed diverse expression profiles under HM, heat, and salt stress, indicating that they might play important roles in the plant response to abiotic stress. For example, *RsCPA09* was significantly up-regulated under HM and heat stress, while exhibited down-regulated under salt stress, indicating that the expression of *RsCPA09* might be a repress factor during salt stress. While *RsCPA13* and *RsCPA31* were all up-regulated under the HM and salt stress, indicating these two genes might play positive roles in response the various abiotic stresses of radish. A recent study in genome-wide identification of the *Gossypium hirsutum NHX* genes showed that most of the *GhNHX* genes were affected by salinity through salt-induced expression patterns analysis [47]. Here, according to the RT–qPCR analysis of *RsNHX* subfamily genes, we found all of them were significantly up-regulated under the 250 mM salt treatment, which indicated that the *RsNHX* genes may be the critical characters for the salt response of radish.

### 3.4. Potential Functions of RsNHXs Genes in Salt Stress

Emerging evidence indicates the Plant NHX proteins play critical roles for salt tolerance through biological function verification. For instance, over-expression of the soybean gene *GmNHX1* in *Arabidopsis thaliana* could enhance salt tolerance through maintaining higher Na^+^ efflux rate and K^+^/Na^+^ ratio, while silencing it may cause soybean plants became more susceptible to salt stress [48]. Similar, over-expression of wheat *TaNHX2* gene in transgenic sunflower improved salinity stress tolerance and growth performance, which showed better growth performance and accumulated higher Na^+^,K^+^ contents in leaves and roots under 200 mM NaCl salt stress [49]. Addtionally, a yeast functional complementation test proved that *GhNHX4A* can partially restore the salt tolerance of the salt-sensitive yeast mutant *AXT3*, while silencing it decreased the resistance of cotton [47]. In the present study, ectopic over-expression and inhibited-expression of the *RsNHX1* gene in *Arabidopsis* significantly affected salt tolerance. Soil culture experiments showed that the growth of *Arabidopsis* OE–*RsNHX1* responded more positively and amiR–*RsNHX1* responded more negatively than the non-transgenic control plants. Furthermore, a TYMV-based VIGS system was used to functionally characterize the *RsNHX1* gene, which was the first time to be employed for silencing the endogenous gene of radish. The expression levels of *RsNHX1* gene were successfully silenced in pTY–*RsNHX1* lines and the seedlings showed more salt damage than controls, which clarified that *RsNHX1* may be a potential regulator in response to salt stress of radish.

According to our results, 60 *CPA* candidate genes of radish were firstly identified on the whole genome level. These genes could be clustered into three subfamilies, including nine *RsNHXs*, ten *RsKEAs*, and 41 *RsCHXs*. 58 genes were mapped to the nine chromosomes based on radish genome sequences. All the 60 *RsCPA* genes had various expression levels in the leaves, roots, cortex, cambium, and xylem at different development stages, as well as under various abiotic stresses. RT–qPCR analysis indicated that all nine *RsNHXs* genes showed upregulated trends after 250 mM NaCl exposure. The *RsCPA31* (*RsNHX1*) gene, which might be the most important members of the RsNHX subfamily, exhibited obvious increased expression levels during 24h salt stress treatment. Heterologous over-expression and inhibited expression of *RsNHX1* in *Arabidopsis* showed that *RsNHX1* had a positive function in salt tolerance. Meanwhile, TYMV-based gene silence system was firstly used to functionally characterize the candidate gene in radish, and the silence of endogenous *RsNHX1* in radish was more susceptible to the salt stress. The results would be useful to understand the complexity of the *RsCPA* gene family and could provide a valuable resource to explore the potential functions of *RsCPA* genes in radish.

## 4. Materials and Methods

### 4.1. Sequence Collection, CPA Identification, and Phylogenetic Analysis

Whole genome sequences of CPA were obtained from the radish genome database (RGD, http://radish-genome.org/) [33]. The CPA protein sequence of *Arabidopsis* was downloaded from TAIR10 (http://www.arabidopsis.org) [50,51]. The CPA protein sequence of *Brassica rapa* was available from *Brassica* database (BRAD, http://brassicadb.org/brad/) [52]. *CPA* family candidate genes with the Na^+^/H^+^ exchanger domain (PF00999) were obtained from the Pfam database (http://pfam.xfam.org) [53]. Then, the Hidden Markov Model (HMM) search was carried out by HMMER 3.0 [54,55]. Subsequently, CDD (https://www.ncbi.nlm.nih.gov/cdd) and InterPro (http://www.ebi.ac.uk/x/pfa/iprscan/) were employed to verify the integrity of the Na^+^/H^+^ exchanger domain [56,57]. In addition, the ExPASy ProtParam (https://www.expasy.org/) was performed to predict the physical and chemical properties of RsCPA proteins, such as the number of amino acids (AA), MW, pI, and instability index [58].

All CPA protein sequences in radish, *Brassica rapa* and *Arabidopsis* were imported to generate the phylogenetic tree using MEGA 6.0 with the neighbor-joining (NJ) and bootstrap value set to 1000 replicates [59,60]. The phylogenetic tree was visualized using Evolview (http://www.evolgenius.info/evolview/) [61].

### 4.2. Gene Structure, Motif Composition, and Promoter Element Analysis

The structure information of *RsCPA* genes were displayed by TBtools (https://github.com/CJ-Chen/TBtools) and the conserved motifs were identified by MEME (http://meme.nbcr.net/meme/tools/meme) [62,63]. Moreover, the 2000 bp upstream sequence (putative promoter region) of all *RsCPA* genes were extracted by TBtools [62]. The *cis*-acting regulatory elements were predicted through PlantCARE (http://bioinformatics.psb.ugent.be/webtools/plantcare/html/) [64]. Additionally, TransMembrane prediction was analyzed by Hidden Markov Models Server v.2.0 (TMHMM, http://www.cbs.dtu.dk/services/TMHMM/) [65] and the protein–protein relationships of stress-inducible *RsCPA* orthologs was evaluated by STRING 9 (https://string-db.org) [66].

### 4.3. Synteny Analysis and Chromosomal Localization

Synteny analysis was performed by the method described in the Plant Duplicate Gene Database (PlantDGD, http://pdgd.njau.edu.cn:8080/) [67]. The collinear block was identified by RsCPA duplication events in the MCScanX [68]. The data were integrated and plotted by using Circos [69]. Based on the annotation information from RGD and duplications of the *RsCPA* genes, the corresponding location distributions of *RsCPA* genes in chromosomes were displayed by TBtools [62].

### 4.4. Expression Analysis of RsCPAs Based on the RNA-Seq. Data

Illumina RNA-Seq. data were downloaded from the NODAI Radish Genome Database and used for the transcriptional profiling of *RsCPA* genes in five tissues (cortical, cambium, xylem, root tip, and leaf) and six stages (7, 14, 20, 40, 60, and 90 days after sowing) [70]. The RPKM method was used to analyze the expression level for each *RsCPA* gene and the heatmap was displayed by TBtools [62]. Furthermore, the expression patterns of *RsCPA* genes during abiotic stress, including heavy metal (HM, such as Cd, Cr and Pb), high temperature, and salt stress were extracted and analyzed from the transcriptome data of radish taproots [71,72,73,74,75].

### 4.5. Plant Material, Salt Stress Treatment, and RT-qPCR Expression Analysis

The radish variety ‘NAU–XBC’ was used, which is an advanced inbred line with white flesh and white skin in taproot, and sensitive to salt stress. The seeds were germinated at 25 °C in the dark for 3 days and then cultivated in the growth chamber with a day/night temperature of 25/18 °C (16/8 h), 60% humidity, and 12,000 lx light. The three-week-old radish seedlings were grown in a plastic container with half-strength Hoagland nutrient solution, as previously described [76]. One week later, seedlings were treated with 250 mM NaCl and the NaCl-free solution was used as a control. Leaves were collected in triplicate at 0, 3, 6, 12, 24, 48, and 96 h after NaCl treatment and immediately frozen in liquid nitrogen and stored at −80 °C. Total RNA was isolated using the TRIzol reagent RNAsimple total RNA kit (Tiangen, Beijing, China) and reverse transcribed into cDNA using the PrimeScript™ RT reagent kit (Takara, Dalian, China) according to the instructions. RT–qPCR analysis was carried out on a LightCycler^®^ 480 System (Roche, Mannheim, Germany). The 2^−^^ΔΔCT^ formula was used to calculate the relative expression level [77]. *RsActin* was regarded as the internal reference gene. The primers used for RT–qPCR are listed in Table S5.

*Arabidopsis thaliana* (ecotype: Col–0) plants were used for heterologous over-expression and inhibited expression *RsNHX1*. In addition, the advanced inbred line ‘NAU–YH’ seedlings were used to perform the VIGS experiment [78].

### 4.6. Genetic Transformation and Generation of RsNHX1 Transgenic Lines in Arabidopsis

To generate over-expression lines, the ORF sequence of *RsNHX1* was cloned into the pCAMBIA2301 vector, using with *Bam*HI/*Kpn*I restriction sites, and driven by a 35S promoter [79]. For amiRNA construction, the natural miR319a sequence as the backbone was exchanged with the *amiRRsNHX1* via the overlapping PCR method [80]. The 418 bp specific sequence was inserted with the *Xba*I and *Sac*I digestion sites and then transferred into the pCAMBIA2301 vector [76]. These fusion plasmids were introduced into *Arabidopsis* using *A. tumefaciens*-mediated transformation with the floral dip method [81,82]. Transgenic lines were screened by ½ Murashige and Skoog (½ MS) solid medium with 100 mg∙L^−1^ kanamycin. Following, the 14-day-old wide type and transgenic line seedlings were planted in sterilized soil one week later and treated with 200 mM NaCl solution every other day [83]. Additionally, the seeds were sown on ½ MS solid media containing 0 and 100 mM NaCl to calculate the germination ratio as well as the root length after two weeks [24].

### 4.7. VIGS-Mediated Silencing of RsNHX1 in Radish

The VIGS experiment was conducted to functionally characterize *RsCPA,* as previously described [78]. The 40 bp specific sequence and its reverse complementary fragment of *RsNHX1* and *RsPDS* were separately synthesized and phosphorylated, and inserted into the pTY–S vector by digestion with *SnaBI* and transformed to obtain positive clones. The primers used for vector construction to identify *RsNHX1*-silenced plants are listed in Table S6. The pTY–S empty vector and self-hybridized palindromic oligonucleotide of *RsPDS*-silencing vector were regarded as negative and positive controls, respectively. Particle bombardment was performed on the two-four fully expanded leaves from ‘NAU–YH’ plants using the PDS–1000/He bio-gun (Bio–Rad, Hercules, CA, USA) to trigger *RsNHX1* silencing [84]. Five plants were bombarded in each experiment. Three weeks later, the inoculated plant leaf phenotype was observed and sampled to analyze the downstream gene level and silencing efficiency. RT–qPCR was used to further confirm *RsNHX1*-silencing. The primers used for RT–qPCR are listed in Table S5. Afterwards, the two positive plants were treated with 250 mM NaCl solution to observe the phenotypes.

## Figures and Tables

**Table 1:** The number of cation proton antiporter (*CPA*) genes in 17 plant species.

Species	CHX Number	NHX Number	KEA Number	Total Gene Number
*Arabidopsis thaliana*	28	8	6	42
*Raphanus sativus*	41	9	10	60
*Brassica rapa*	40	11	13	64
*Pyrus bretschneideri*	27	14	12	53
*Malus domestica*	42	12	7	61
*Prunus persica*	26	6	5	37
*Fragaria vesca*	24	6	5	35
*Prunus mume*	23	7	4	34
*Vitis vinifera*	17	8	4	29
*Oryza sativa*	18	8	4	30
*Zea mays*	16	11	6	33
*Sorghum bicolor*	17	7	4	28
*Selaginella*	3	7	4	14
*Ostreococcus*	0	6	4	10
*Chlorophyta reinhardtii*	0	9	3	12
*Physcomitrella patens*	5	10	7	22
*Populus trichocarpa*	29	8	7	44

## Supplementary Materials

Supplementary Materials can be found at https://www.mdpi.com/1422-0067/21/21/8262/s1. Figure S1. LOGO of 20 amino acid motifs in RsCPA proteins. Figure S2. Number of *cis*–acting elements on promoters of *RsCPA* genes. Figure S3. Functional interaction networks of 60 RsCPA proteins. Figure S4. RNA–Seq of *RsCPA* genes under different treatments in the radish taproot. Figure S5. The expression levels of *RsNHX* genes at different times under 250 mM NaCl treatment. Figure S6. PCR analysis of over–expression and inhibited–expression in T_3_ transgenic *Arabidopsis* plants. Table S1. Basic data for the CPA proteins in radish. Table S2. Number of transmembrane regions in RsCPA proteins. Table S3. Synteny blocks of *CPA* genes between radish and *Arabidopsis* genomes. Table S4. Ka/Ks analysis of 24 *RsCPA* duplicated gene pairs. Table S5. Specific primers of *RsCPA* genes for qRT–PCR. Table S6. Specific primers of *RsNHX1* for vector construction.

## Abbreviations

AA	Amino Acids
ABA	Abscisic Acid
AtCPA	*Arabidopsis thaliana Cation proton antiporter* gene
BraCPA	*Brassica rapa Cation proton antiporter* gene
BRAD	Brassica database
Cd	Cadmium
Chr(s)	Chromosome(s)
CHX	Cation/H^+^ exchanger
CPA	Cation proton antiporter
Cr	Chromium
DD	Dispersed duplication
GA_3_	Gibberellin A_3_
GRAVY	Grand average of hydropathicity
HM	Heavy metal
HMM	Hidden Markov Model
KEA	K^+^ efflux antiporter
MCScanX	Multiple Collinearity Scan toolkit
MeJA	Methyl Jasmonate
MEME	Multiple Em for Motif Elicitation
MW	Molecular weight
NHX	Na^+^/H^+^ exchanger family
NJ	Neigbor-joining
ORF	Open reading frame
Pb	Lead
PD	Proximal duplicatio
pI	Theoretical isoelectric point
PlantDGD	Plant Duplicate Gene Database
RGD	Radish Genome Database
RPKM	Reads per kilobase per million reads
RsCPA	*Raphanus sativus Cation proton antiporter* gene
RT–qPCR	Real-Time Quantitative Polymerase Chain Reaction
TD	Tandem duplication
TMHMM	TransMembrane prediction was analyzed by Hidden Markov Models
TRD	Transposed duplication
TYMV	Turnip yellow mosaic virus
UTR	Untranslated region
VIGS	Virus-induced gene silencing
WGD	Whole-genome duplication
WT	Wide type
