# Supplementary figures and images for: Genome-Wide Identification and Functional Characterization of the Cation Proton Antiporter (CPA) Family Related to Salt Stress Response in Radish (Raphanus sativus L.)
Source: Int J Mol Sci. 2020 Nov 4;21(21):8262. doi: 10.3390/ijms21218262 (PMC7662821; doi:10.3390/ijms21218262)

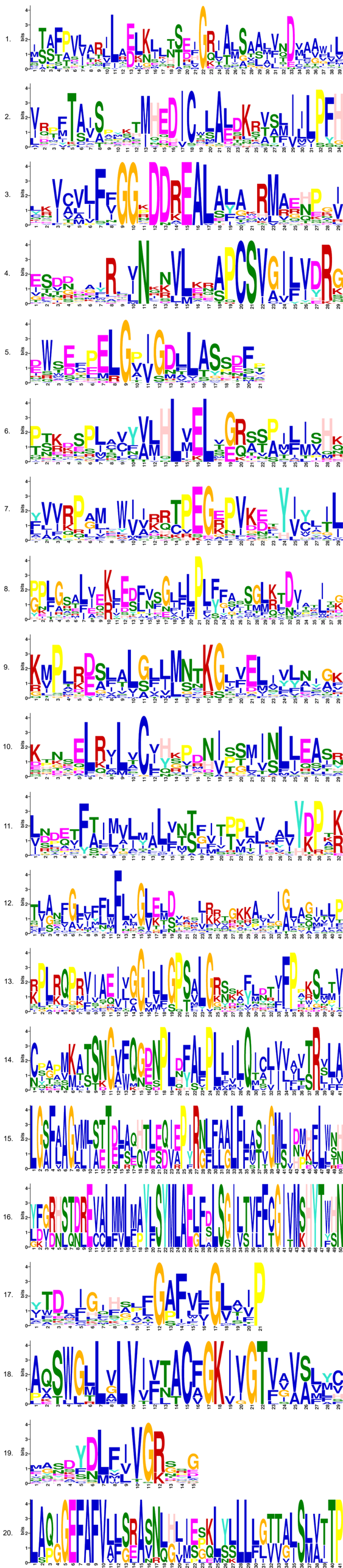

Supplement: Supplementary file 1 [file ijms-21-08262-s001.zip › supplementary materials/Figure S1. The LOGO of 20 amino acid motifs in CPA proteins..pdf]

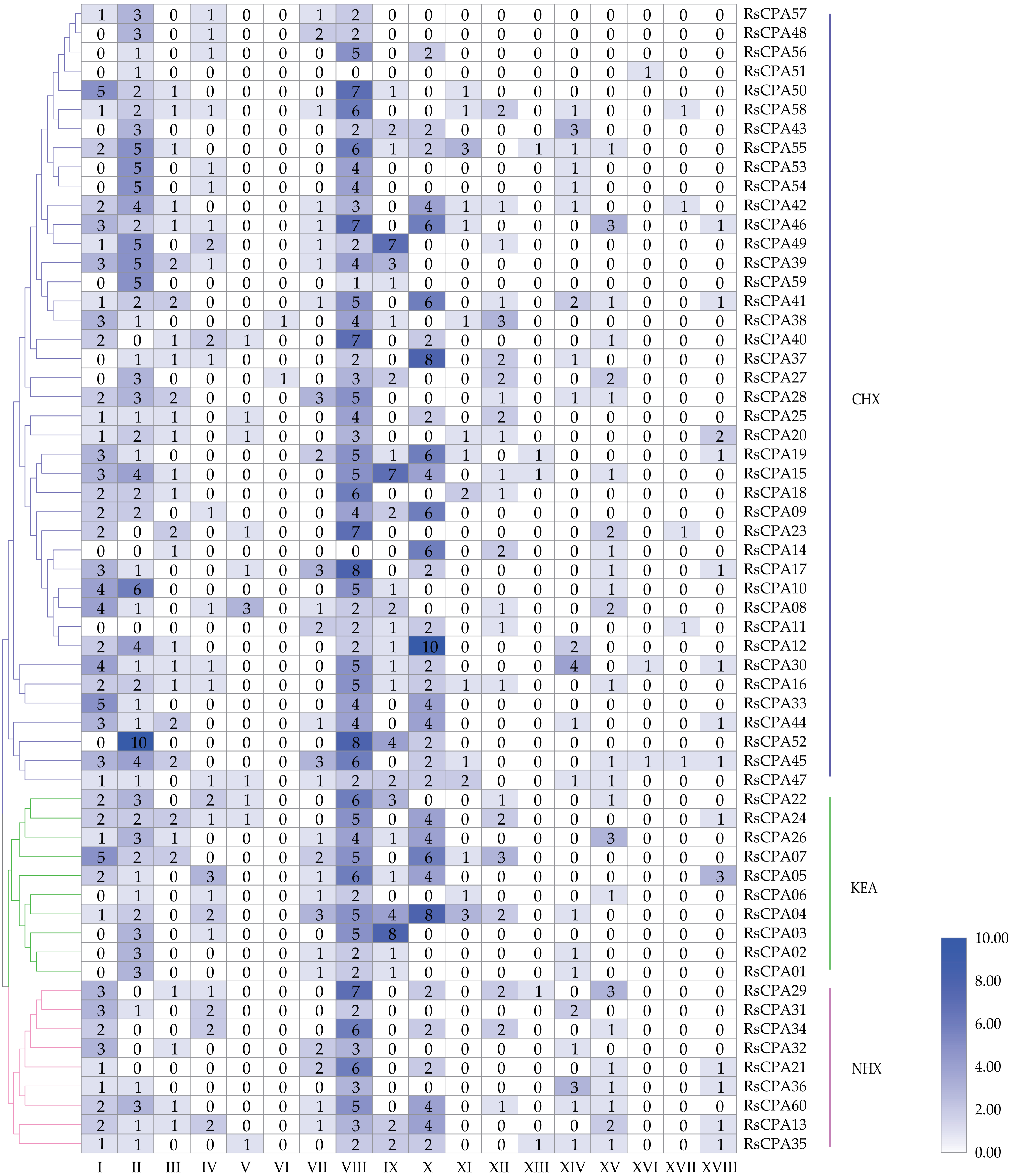

Supplement: Supplementary file 1 [file ijms-21-08262-s001.zip › supplementary materials/Figure S2. Number of cis–acting elements on promoters of RsCPA genes..pdf]

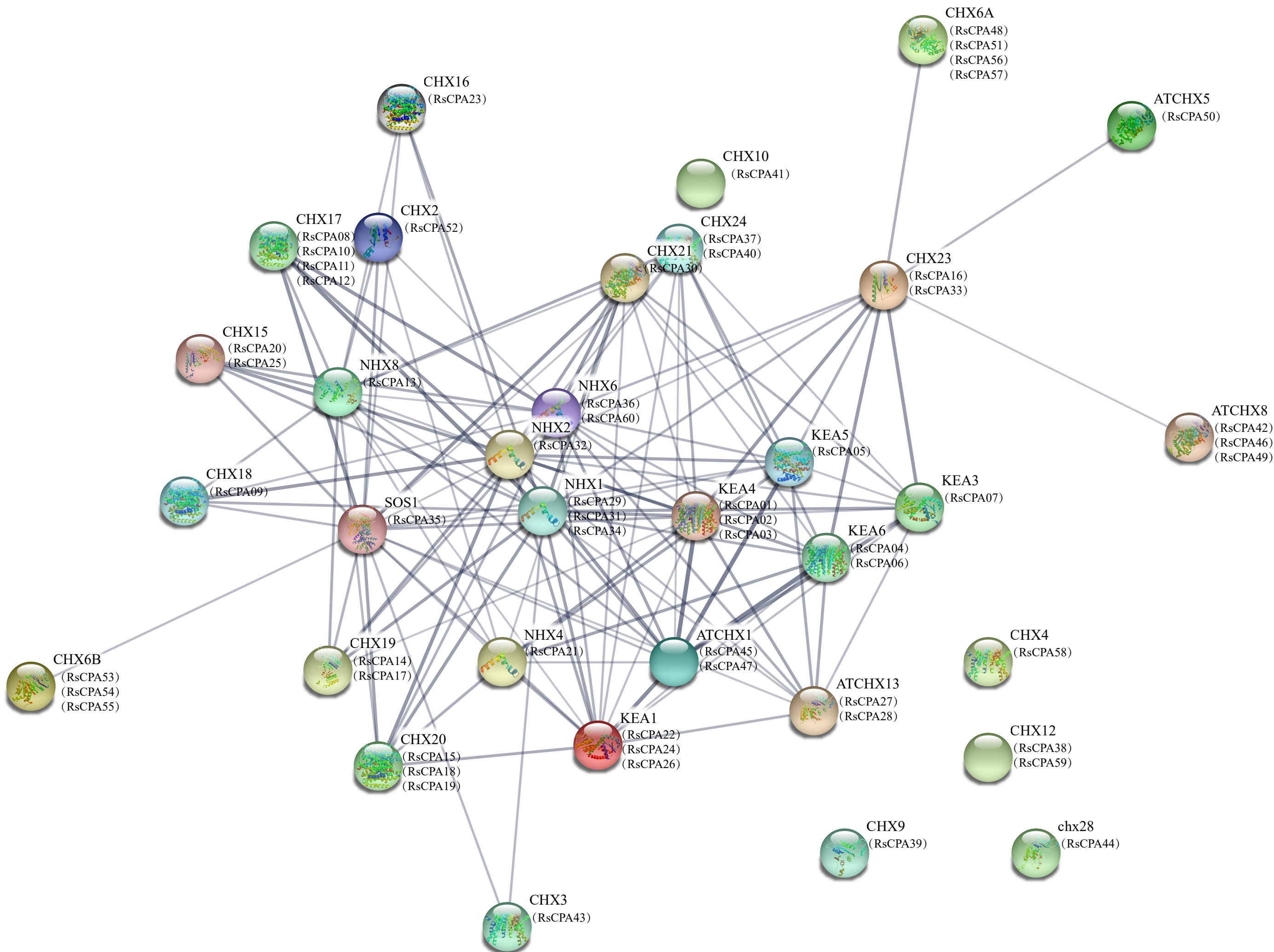

Supplement: Supplementary file 1 [file ijms-21-08262-s001.zip › supplementary materials/Figure S3. Functional interaction networks of 60 RsCPA proteins..pdf]

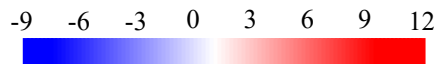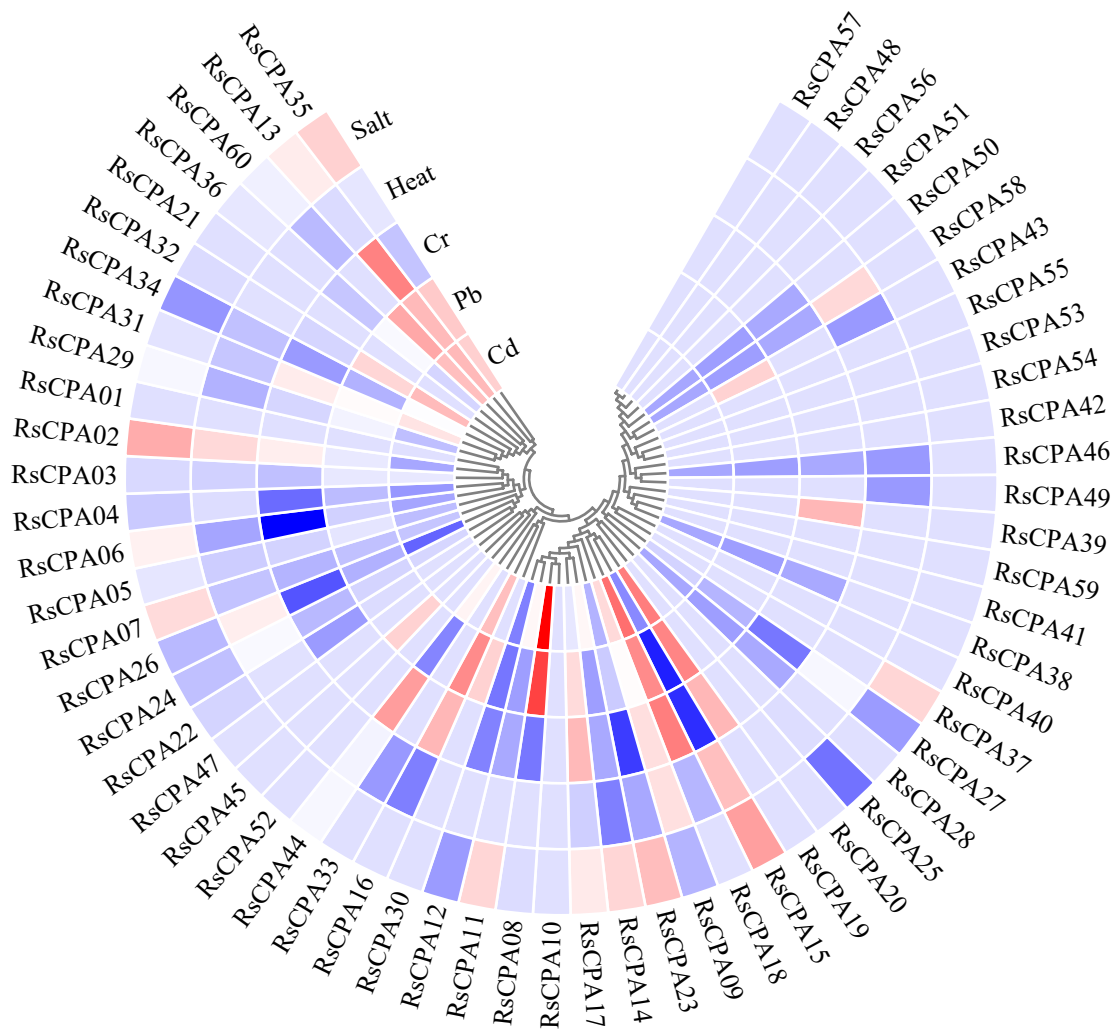

Supplement: Supplementary file 1 [file ijms-21-08262-s001.zip › supplementary materials/Figure S4. The RNA-Seq of RsCPA genes under different treatments in radish taproot..pdf]

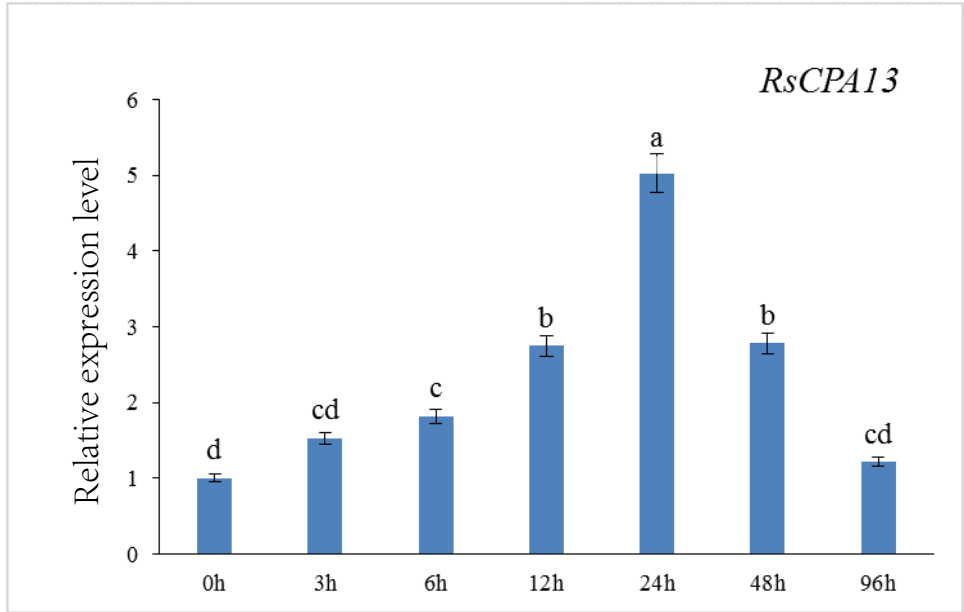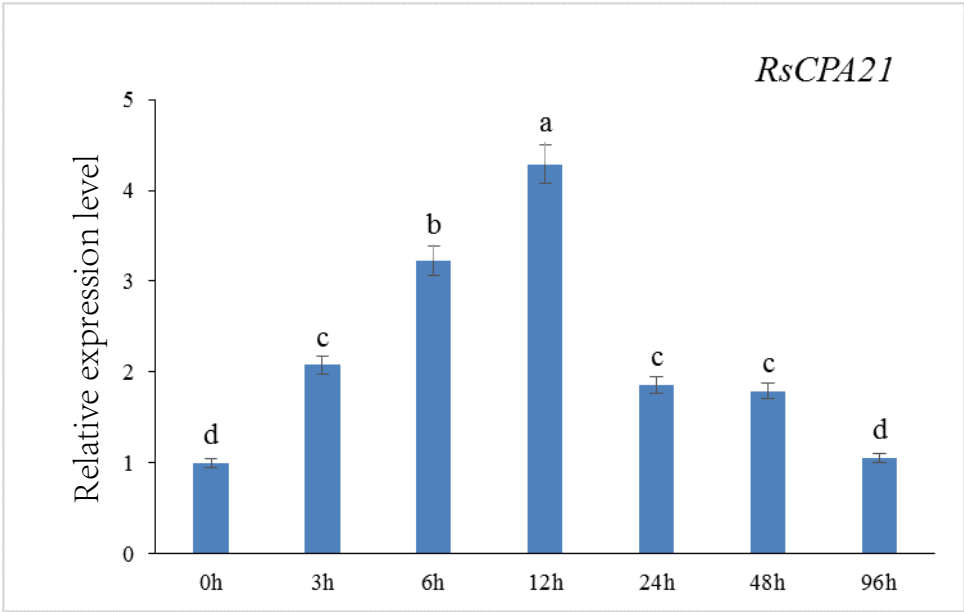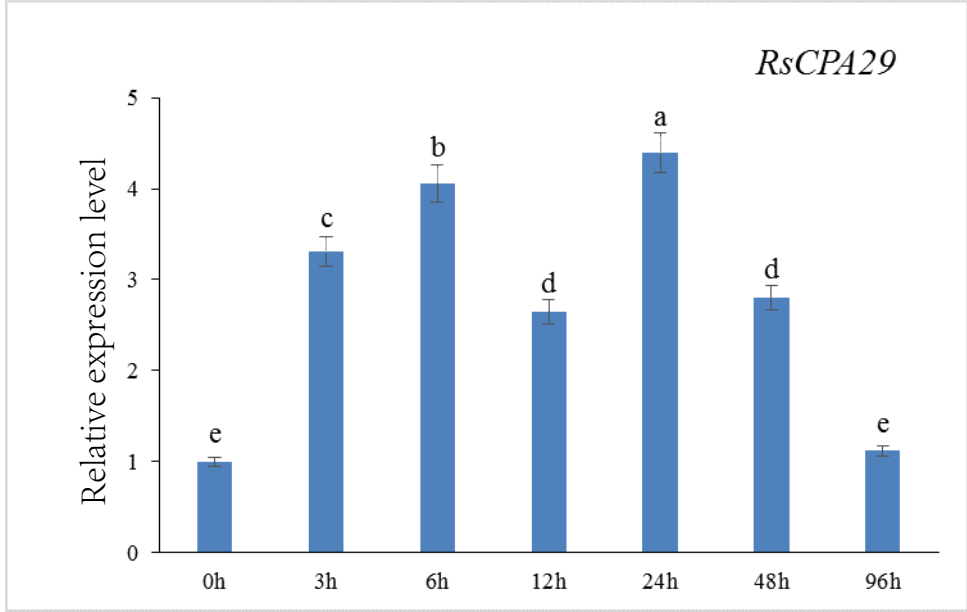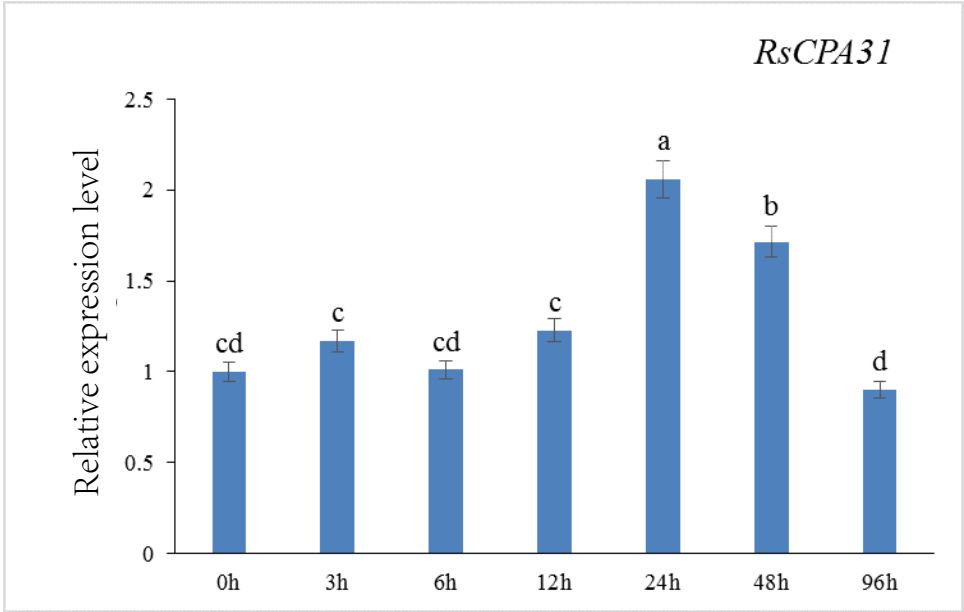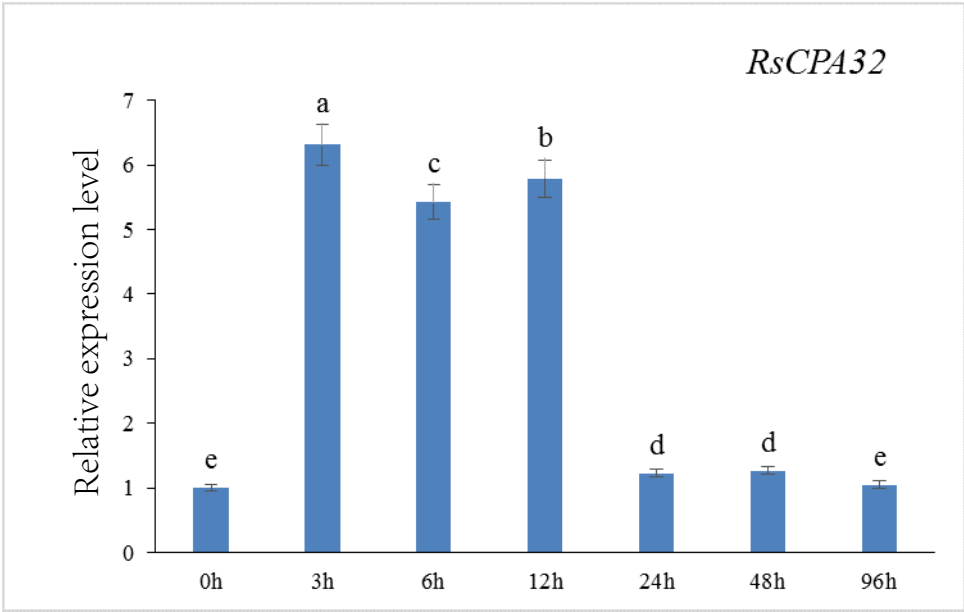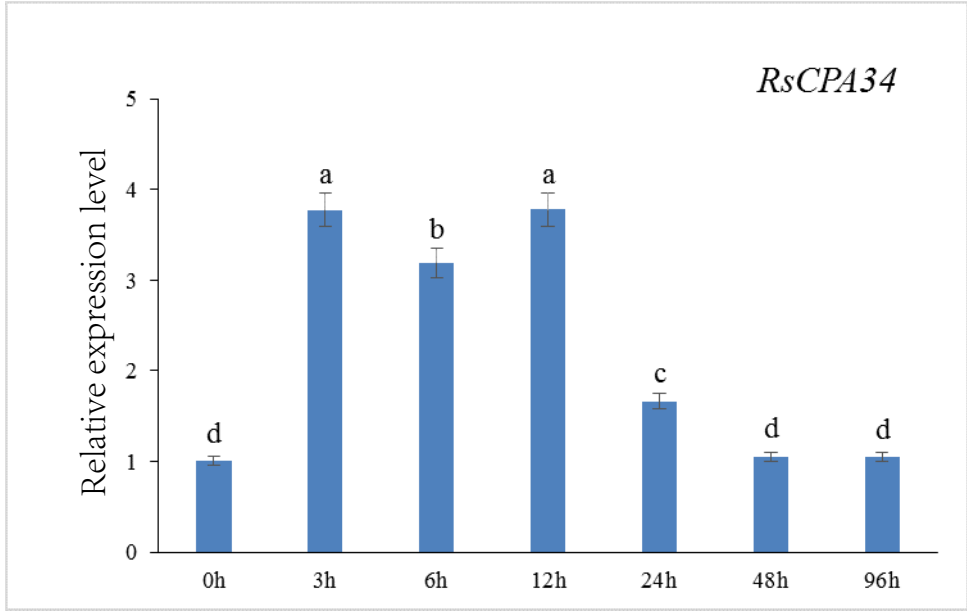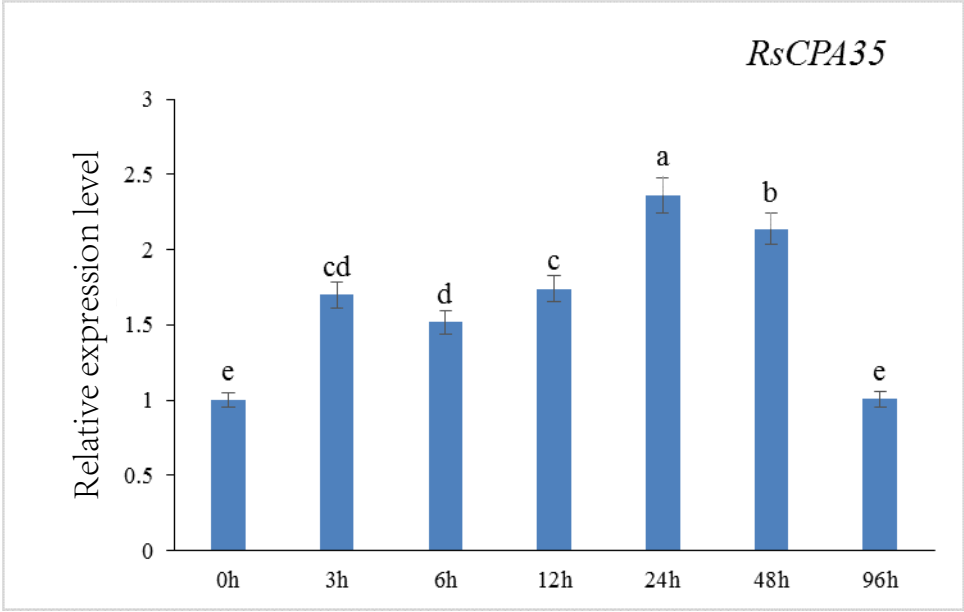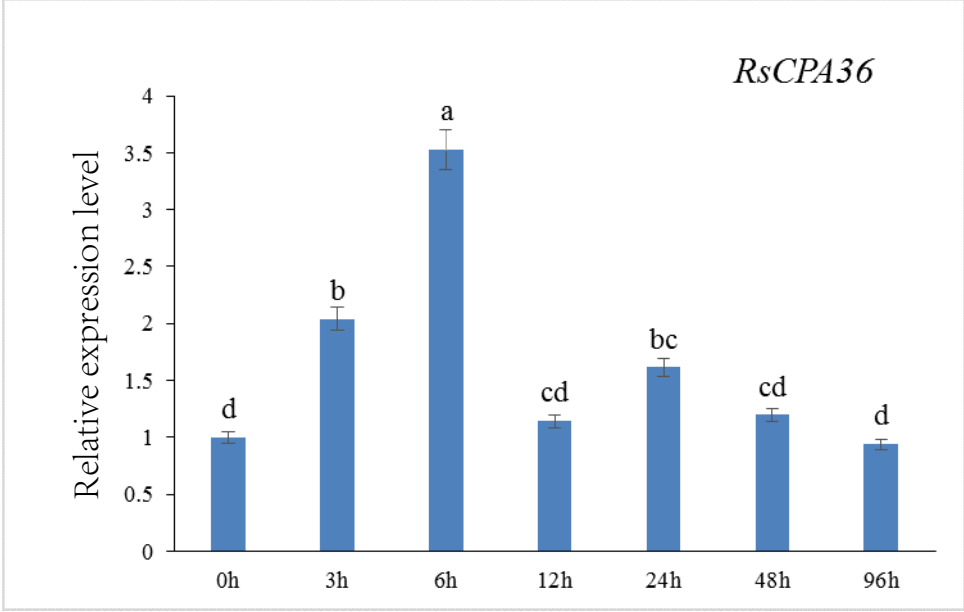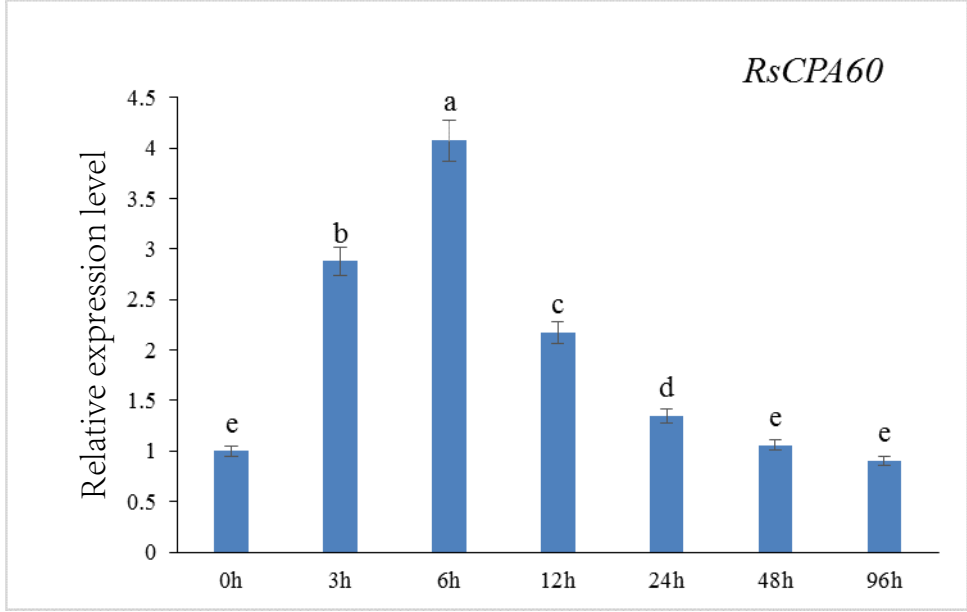

Supplement: Supplementary file 1 [file ijms-21-08262-s001.zip › supplementary materials/Figure S5. The expression levels of RsNHX genes at different times under 250 mM NaCl treatment..pdf]

(a)

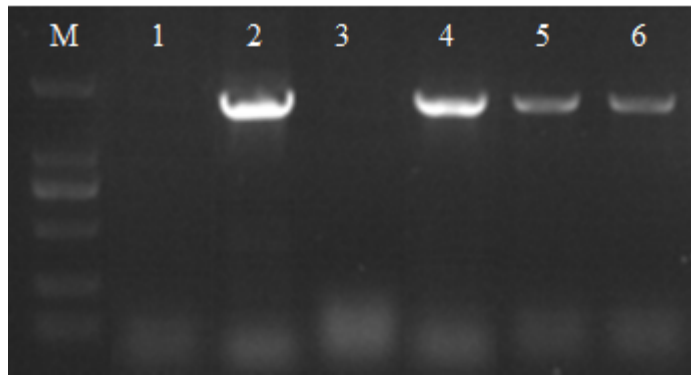

(b)

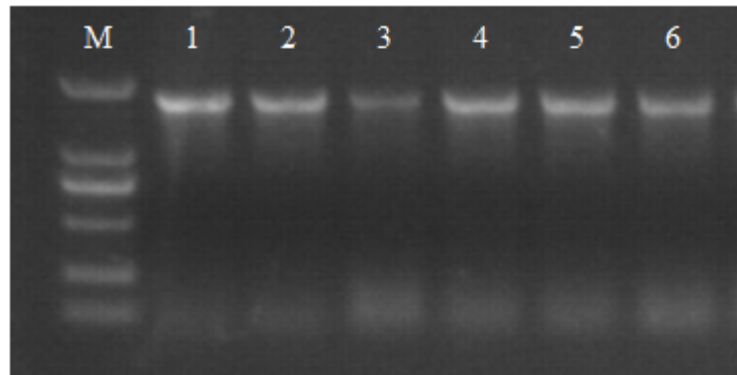

Supplement: Supplementary file 1 [file ijms-21-08262-s001.zip › supplementary materials/Figure S6. PCR analysis of over-expression and inhibited-experssion T3 transgenic Arabidopsis plants..pdf]
